# Prevalence trend and burden of neglected parasitic diseases in China from 1990 to 2019: findings from global burden of disease study

**DOI:** 10.3389/fpubh.2023.1077723

**Published:** 2023-05-24

**Authors:** Yi Xie, Dandan Shi, Xu Wang, Yayi Guan, Weiping Wu, Ying Wang

**Affiliations:** National Institute of Parasitic Diseases, Chinese Center for Disease Control and Prevention (Chinese Center for Tropical Diseases Research), NHC Key Laboratory of Parasite and Vector Biology, WHO Collaborating Centre for Tropical Diseases, National Center for International Research on Tropical Diseases, Shanghai, China

**Keywords:** neglected tropical diseases, parasitic diseases, GBD, disease burden, prevalence

## Abstract

**Objective:**

This study sought to investigate the parasitic diseases of neglected tropical diseases defined by the World Health Organization based on the Global Burden of Disease Study (GBD) database. Importantly, we analyzed the prevalence and burden of these diseases in China from 1990 to 2019 to provide valuable information to formulate more effective measures for their management and prevention.

**Methods:**

Data on the prevalence and burden of neglected parasitic diseases in China from 1990 to 2019 were extracted from the global health data exchange (GHDx) database, including the absolute number of prevalence, age-standardized prevalence rate, disability-adjusted life year (DALY) and age-standardized DALY rate. Descriptive analysis was used to analyze the prevalence and burden changes, sex and age distribution of various parasitic diseases from 1990 to 2019. A time series model [Auto-Regressive Integrated Moving Average (ARIMA)] was used to predict the DALYs of neglected parasitic diseases in China from 2020 to 2030.

**Results:**

In 2019, the number of neglected parasitic diseases in China was 152518062, the age-standardized prevalence was 11614.1 (95% uncertainty interval (UI) 8758.5–15244.5), the DALYs were 955722, and the age-standardized DALY rate was 54.9 (95% UI 26.0–101.8). Among these, the age-standardized prevalence of soil-derived helminthiasis was the highest (9370.2/100,000), followed by food-borne trematodiases (1502.3/100,000) and schistosomiasis (707.1/100,000). The highest age-standardized DALY rate was for food-borne trematodiases (36.0/100,000), followed by cysticercosis (7.9/100,000) and soil-derived helminthiasis (5.6/100,000). Higher prevalence and disease burden were observed in men and the upper age group. From 1990 to 2019, the number of neglected parasitic diseases in China decreased by 30.4%, resulting in a decline in DALYs of 27.3%. The age-standardized DALY rates of most diseases were decreased, especially for soil-derived helminthiasis, schistosomiasis and food-borne trematodiases. The ARIMA prediction model showed that the disease burden of echinococcosis and cysticercosis exhibited an increasing trend, highlighting the need for further prevention and control.

**Conclusion:**

Although the prevalence and disease burden of neglected parasitic diseases in China have decreased, many issues remain to be addressed. More efforts should be undertaken to improve the prevention and control strategies for different parasitic diseases. The government should prioritize multisectoral integrated control and surveillance measures to prioritize the prevention and control of diseases with a high burden of disease. In addition, the older adult population and men need to pay more attention.

## 1. Introduction

Neglected tropical diseases (NTDs) represent a group of very diverse diseases caused by bacteria, viruses, worms, ectoparasites, fungi, protozoa or non-infectious causes. In 2010, the World Health Organization (WHO) established a list of 17 diseases as NTDs (1). Since 2016, three diseases have been added to this list, which now includes 20 non-communicable diseases or non-communicable disease groups (2). Among the neglected tropical diseases, there are 11 parasitic diseases (hereinafter referred to as neglected parasitic diseases), including cysticercosis, cystic echinococcosis, dracunculiasis, food-borne trematodiases, lymphatic filariasis, soil-derived helminthiasis, schistosomiasis, onchocerciasis, Chagas disease, leishmaniasis and African trypanosomiasis (3). It is well-established that neglected parasitic diseases are often challenging to control. Neglected parasitic diseases are a severe global public health problem. These diseases are transmitted by vectors and spread back and forth between animals and humans, increasing the risk of suffering from these diseases. Livestock keepers are the most susceptible population, given that they are in close contact with livestock and have the least access to health services and information (4).

China was once the country with the most severe prevalence of parasitic diseases, with malaria, schistosomiasis, filariasis, and other serious hazards to human health. Since the founding of the People’s Republic of China, the government has attached great importance to the prevention and control of parasitic diseases and has made considerable progress in controlling and eliminating neglected parasitic diseases in China. Over the past 70 years, the prevalence and transmission of neglected parasitic diseases in China have been effectively controlled. The elimination of lymphatic filariasis and malaria in China were certified by WHO in 2007 and 2021, respectively (5). However, some parasitic diseases still present transmission risks and can significantly burden our economy, highlighting the need to improve current prevention and control strategies. Previous studies on parasitic diseases in China were mostly focused on surveys of prevalence or infection rates, while studies on the burden of disease were mostly limited to some regions and provinces, with few systematic national analyses. The Global Burden of Disease (GBD) is a global disease related data analyzed and released by the Institute for Health Metrics and Evaluation (IHME) of the University of Washington, US. It provides a comprehensive assessment of disease burden caused by 369 diseases and injuries and 87 risk factors in 204 countries and regions worldwide. GBD is currently the most reliable and widely covered disease burden database in the world. Based on the GBD database, this study analyzed the trend and burden of neglected parasitic diseases in China from 1990 to 2019 and predicted the disease burden of neglected parasitic diseases in China in the next 10 years to provide essential information for further prevention and control of neglected parasitic diseases in China.

## 2. Method

### 2.1. Data source

Data was collected from the “Global Burden of Disease 2019, GBD 2019.” Over the years, many studies have been conducted on the initiation, design, data collection and standardized analysis, statistical model construction and index calculation generation based on the GBD database. Briefly, the Global Burden of Disease Study 2019 estimates the disease burden caused by 369 diseases and injuries in 204 countries and regions using standard and reproducible methods and reports them by country and region, year, sex and age group (6). Detailed disease data can be downloaded from the global health data exchange (GHDx) website.^1^ GBD provides high-quality data with internal consistency and comparability and is currently an internationally recognized evaluation system. Data on China mainly emanate from the national population census, disease monitoring point system, maternal and child health monitoring system, death cause reporting system of China Center for Disease Control and Prevention, tumor registration data, and relevant literature and research reports on the incidence and prevalence of various diseases. For a detailed introduction and usage of GBD 2019, please refer to the following references (7–9).

### 2.2. Definition of neglected parasitic diseases

The GBD 2019 cause list hierarchy is organized into four levels of causes that are mutually exclusive and collectively exhaustive. Level 1 has three broad categories: communicable, maternal, neonatal, and nutritional (CMNN) disorders; non-communicable diseases (NCDs); and injuries. Level 2 has 21 cause groups, such as neoplasms and cardiovascular diseases. Levels 3 and 4 are disaggregated in 168 and 276 causes, respectively. NTD-related causes are included in the level 2 group “Neglected tropical diseases and malaria,” which consists of 20 infectious and parasitic diseases, including malaria, NTDs prioritized by the WHO (15 of the 18 NTDs in 2016), and other neglected diseases. In the present study, we included the estimates of six neglected parasitic diseases in GBD in 2019 (10, 11). These diseases are part of the official priority list of WHO and are endemic diseases in China: cystic echinococcosis (mainly caused by *Echinococcus granulosus*), cysticercosis (mainly caused by *Taenia solium, Taenia saginataGoeze*), food-borne trematodiases (mainly caused by *Clonorchis sinensis, Opisthorchis viverrini, Fasciola hepatica, Paragonimus* spp), leishmaniasis (mainly caused by *Leishmania donovani, Leishmania infantum*), schistosomiasis (mainly caused by *Schistosoma japonicum*), and soil-derived helminthiasis (mainly caused by *Ascaris lumbricoides, Trichuris trichura, Ancylostoma duodenale*).

### 2.3. Disease burden indicators

The disability-adjusted life year (DALY) is a widely acknowledged time-based measure for assessing the burden of disease, which can more comprehensively consider the early death and disability burden. It is estimated by the sum of the years of life lost due to premature mortality (YLLs) and years lived with disability (YLDs) for a given disease or injury (12). One DALY represents the loss of 1 year’s healthy life (11). In this study, DALYs were used to quantify the disease burden.

### 2.4. Statistical analysis

The ARIMA model is a differential integrated moving average autoregressive model, also known as an integrated moving average autoregressive model, which is one of the time series forecasting analysis methods. In ARIMA (p, d, q), AR is “autoregressive,” and p is the number of autoregressive terms. MA is the “moving average,” q is the number of terms in the moving average, and d is the number of differences (order) made to make it a stationary sequence (13). This study used the ARIMA model to analyze the trend of disease burden based on DALYs and predicted the disease burden of different neglected parasitic diseases in China from 2020 to 2030.

The incidence and burden of neglected parasitic diseases from 1990 to 2019 were expressed as the absolute number and age-standardized rate (per 100,000). DALYs and 95% uncertainty interval (UI) of neglected parasitic diseases in China were analyzed after stratifying by sex, age and year and Microsoft Excel 2019 was used to collate the data. In this study, the age group (<5, 5–9, 10–14…, 80+) is extracted from the GBD 2019 database, divided into 17 age groups. A descriptive analysis was conducted on the prevalence of various parasitic diseases and DALYs in China in 1990 and 2019 and the sex and age distribution of various parasitic diseases in 2019. The ARIMA model was used to predict and analyze the trend of DALYs of neglected parasitic diseases in China from 2020 to 2030. All analyses and data visualization were performed using R version 4.2.1.

## 3. Results

### 3.1. The prevalence and burden of neglected parasitic diseases in China from 1990 to 2019

**Figure 1:**
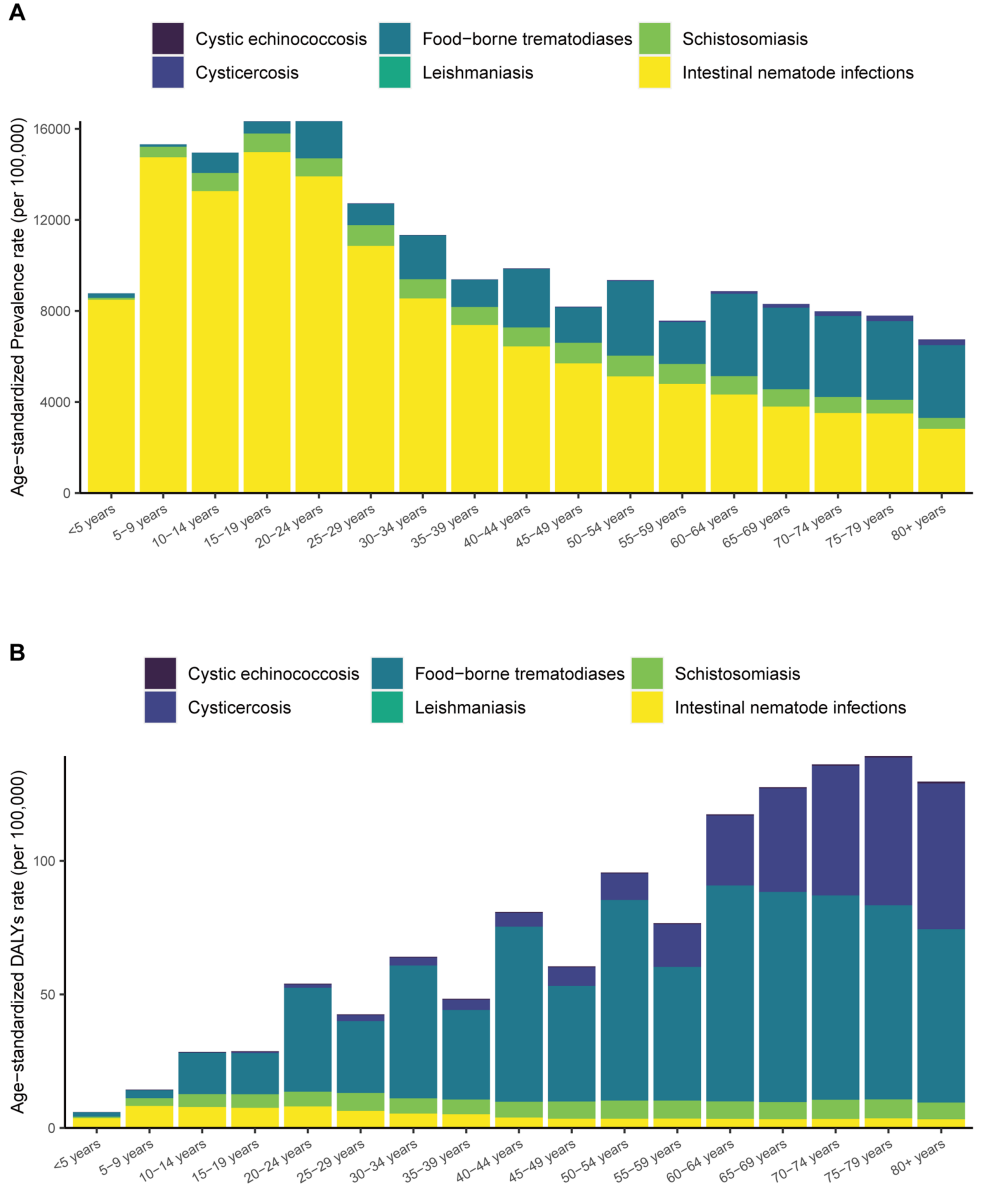
Trends in the age-standardized prevalence and DALY rate of neglected parasitic diseases in China from 1990 to 2019.

Overall, China’s prevalence of neglected parasitic diseases exhibited a downward trend from 1990 to 2019. China’s overall prevalence of neglected parasitic diseases decreased from 331.08 million in 1990 to 152.5 million in 2019. In this respect, the prevalence of cysticercosis exhibited the most significant increase (from 390,000 in 1990 to 640,000 in 2019), and soil-derived helminthiasis decreased the most (from 297.8 million in 1990 to 114.3 million in 2019) (Table 1). The prevalence of cystic echinococcosis did not change significantly, the age-standardized prevalence rate of cystic echinococcosis increased from 1.8/100,000 in 1990 to 2.1/100,000 in 2019, an increase of 16.7%. The prevalence of cysticercosis showed a downward trend, and the age-standardized prevalence rate decreased from 42.7 per 100,000 in 1990 to 32.4 per 100,000 in 2019. Similarly, the prevalence rate of food-borne trematodiases generally showed a downward trend but fluctuated slightly from 2010 to 2015, and the age-standardized prevalence rate decreased from 3001.7/100,000 to 1502.3/100,000. The prevalence of schistosomiasis continued to decline, and the age-standardized prevalence rate decreased from 1349.6 per 100,000 to 707.1 per 100,000. Although a slight increase in prevalence was observed from 2005 to 2017, soil-derived helminthiasis exhibited a downward, and the age-standardized prevalence rate decreased from 241608/100,000 to 93702/100,000 (Figure 1A).

**Table 1:** Prevalence and DALYs of six neglected parasitic diseases in China in 1990 and 2019.

Items	Prevalence						DALYs					
	Number (95% UI)			ASR Per 100,000			Number (95% UI)			ASR Per 100,000		
	1990	2019	Change (%)	1990	2019	Change (%)	1990	2019	Change (%)	1990	2019	Change (%)
Cystic echinococcosis	22711.0 (6656.9–56832.8)	31873.3 (13490–64284.9)	40.3	1.8 (0.6–4.2)	2.1 (0.8–4.5)	12.5	5235.8 (3729.9–8092.9)	4258.6 (2779.1–7095)	−18.7	0.5 (0.3–0.7)	0.3 (0.2–0.5)	−42.3
Cysticercosis	385330.6 (269995.8–538496.5)	642141.3 (418603.5–935001)	66.6	42.7 (30.7–59.1)	32.4 (21.7–47)	−24.1	127875.6 (76217.3–190741.1)	156853 (85033.6–251033.7)	22.7	13.9 (8.4–20.3)	7.9 (4.4–12.5)	−43.0
Food-borne trematodiases	32909352.2 (27847936.7–39242723.9)	26764860.9 (22975782.8–31155876.4)	−18.7	3001.7 (2548.1–3565.8)	1502.3 (1291.2–1741.4)	−50.0	884730.6 (308126.1–1851785.2)	643836.4 (283348–1245718.3)	−27.2	79.7 (28.2–167.3)	36.0 (15.9–69.9)	−54.8
Leishmaniasis	173.4 (34–368.8)	0 (0–0)	−100	0 (0–0)	0 (0–0)	−47.6	83.2 (49–138.6)	19.7 (11.6–32.5)	−76.3	0 (0–0)	0 (0–0)	−75.6
Schistosomiasis	16568270.8 (14041800.7–23153473.2)	10807402.8 (9520954.3–14058672.9)	−34.8	1349.6 (1146.3–1903.3)	707.1 (621.4–929.1)	−100.	199617.9 (132360.9–306929.6)	79764.6 (40520.4–148372.9)	−60.0	17.4 (12–26)	5.1 (2.5–9.5)	−71.0
Intestinal nematode infections / Soil-derived helminthiasis	297764739.6 (233791216.9–367720399.7)	114271784.0 (83663788.3–152149882.7)	−61.6	24160.8 (18955.2–29699.9)	9370.2 (6823.4–12522.5)	−61.2	863177 (474180.6–1428647)	70989.7 (38580.7–120679.2)	−91.8	69.3 (38.2–114.6)	5.6 (3–9.4)	−92.0

From 1990 to 2019, the disease burden of neglected parasitic diseases in China showed a downward trend. DALYs caused by neglected parasitic diseases in China exhibited a downward trend from 1990 (2.1 million) to 2019 (1.0 million), with a decline of 54.0% (Table 1). For instance, the disease burden of cystic echinococcosis showed a downward trend, and the age-standardized DALY rate decreased from 0.5/100,000 to 0.3/100,000. Moreover, the disease burden of cysticercosis generally showed a downward trend, rising slightly from 1990 to 1994 and declining after 1994, while the age-standardized DALY rate decreased from 13.9/100,000 to 7.9/100,000. The disease burden of food-borne trematodiases generally showed a downward trend, but it fluctuated slightly from 2010 to 2015, and the age-standardized prevalence rate decreased from 79.7 per 100,000 to 3600 per 100,000. The disease burden of schistosomiasis continued to decline, and the age-standardized disease burden decreased from 17.4/100,000 to 5.1/100,000. The overall disease burden of soil-derived helminthiasis showed a downward trend, rising slightly from 2005 to 2017, and the age-standardized DALY rate decreased from 69.3 per 100,000 to 5.6 per 100,000 (Figure 1B).

### 3.2. Sex and age distribution of neglected parasitic diseases in China in 2019

**Figure 2:**
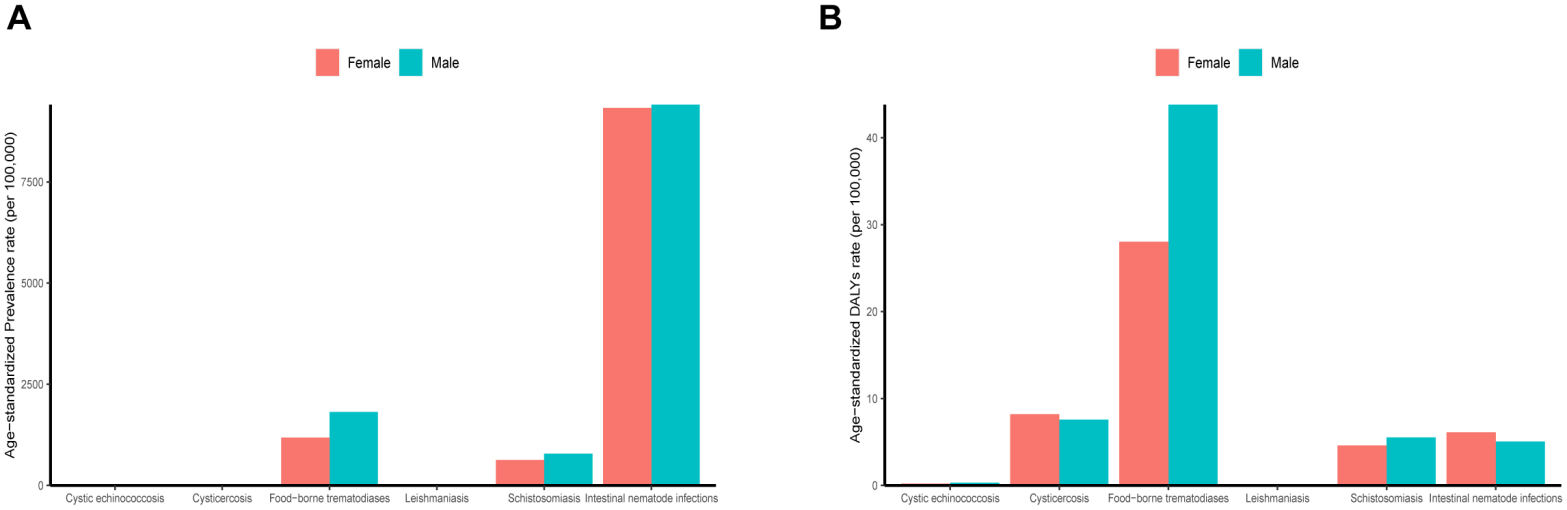
Age-standardized prevalence and DALY rates of neglected parasitic diseases in China by sex in 2019.

From the perspective of sex distribution, the total number of cases of neglected parasitic diseases in China was higher in males (22.7 million cases) than in females (15.5 million cases) in 2019. Compared to females, males exhibited a slightly higher age-standardized prevalence rate (2.2 per 100,000 vs. 1.9 per 100,000) and age-standardized DALY rate (0.3 per 100,000 vs. 0.3 per 100,000) for cystic echinococcosis. Moreover, the prevalence rate (30.6 per 100,000 vs. 34.0 per 100,000) and the age-standardized DALYs rate (10.1 per 100,000 vs. 12.0 per 100,000) of cysticercosis in males was lower than in females. Consistently, the prevalence (181584.0/100,000 vs.118289.0/100,000) and the age-standardized DALYs rate (5430.0/100,000 vs. 3588.0/100,000) of food-borne trematodiases in males was significantly higher than in females. Moreover, the prevalence (783.0/100,000 vs. 628.4/100,000) and the age-standardized DALYs rate (6.2/100,000 vs. 5.1/100,000) of schistosomiasis in males was higher than in females. The age-standardized prevalence rate of leishmaniasis was 0. The age-standardized DALYs rate in males (0.002/100,000) was higher than in females (0.000/100,000). The age-standardized prevalence rate of soil-derived helminthiasis in males was higher than in females (94.1/100,000 vs. 93.3/100,000). However, the age-standardized DALYs rate in males (5.0/100,000) was lower than in females (6.1/100,000) (Figure 2; Supplementary Table S1).

**Figure 3:**
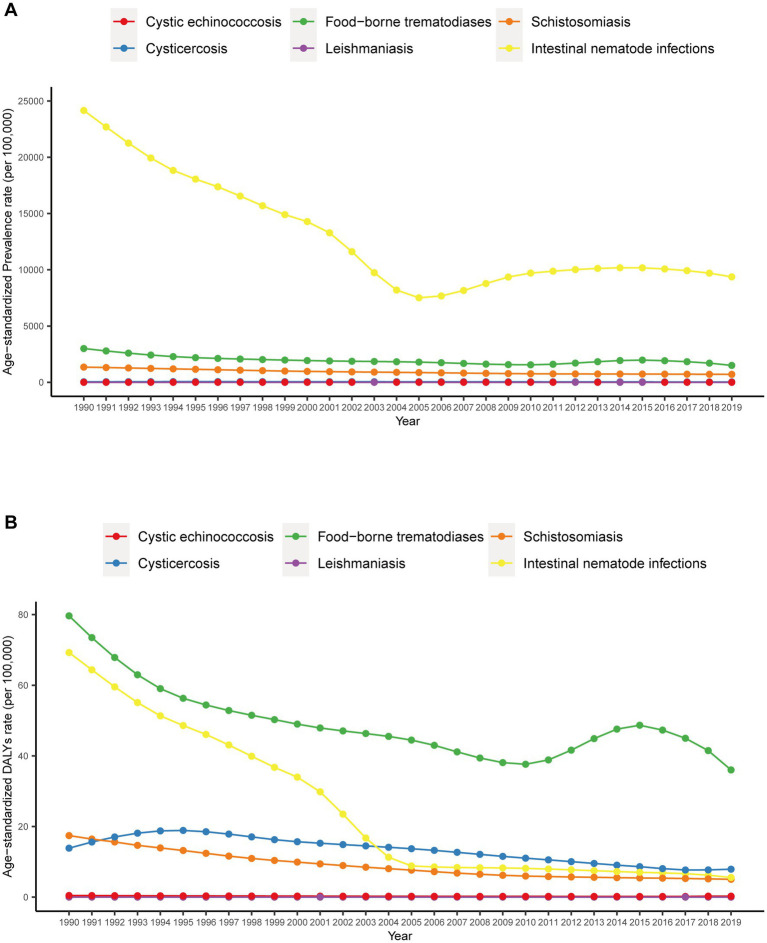
Age-standardized prevalence and DALY rate of neglected parasitic diseases in China by age in 2019.

In this study, the age group (<5, 5–9, 10–14…, 80+) is extracted from the GBD 2019 database, divided into 17 age groups. In terms of prevalence, the age-standardized prevalence of neglected parasitic diseases in China decreased with age in 2019. The age-standardized prevalence rate of cystic echinococcosis did not change significantly with age, and the age-standardized prevalence rate of the “20–24 years” group was the highest. The prevalence rate of cysticercosis increased with age, and the age-standardized prevalence rate of the “≥ 80 years” group was the highest. The prevalence of food-borne trematodiases fluctuated with age, and the age-standardized prevalence rate of the “60–64 years” group was the highest. Schistosomiasis was evenly distributed in other groups except for the “<5 years” group. The distribution of soil-derived helminthiasis showed a positive skewness with age, and the peak was observed in the “15–19 years” group. From the perspective of disease burden, the total disease burden of parasitic diseases increased with age. The change in disease burden of various diseases with age was consistent with disease prevalence. The age-standardized DALY rate of taeniasis was the most prominent with increased age (Figure 3; Supplementary Figures S1, S2).

### 3.3. Prediction of the burden of neglected parasitic diseases in China using the ARIMA model

**Figure 4:**
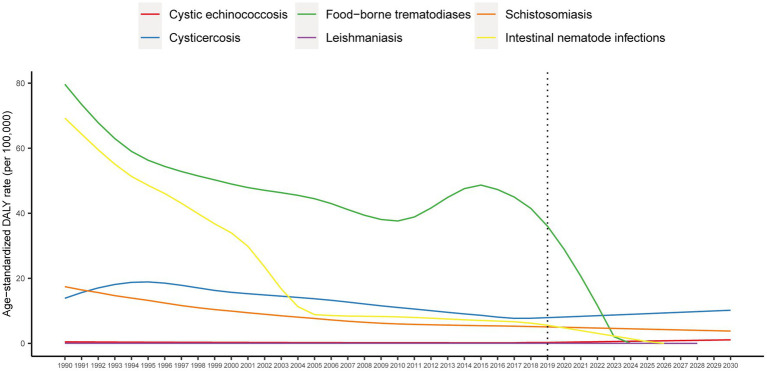
Age-standardized DALY rate prediction trend of neglected parasitic diseases in China from 2020 to 2030.

This study used the ARIMA model to predict the disease burden of neglected parasitic diseases in China from 2020 to 2030. Figure 4 shows the time series of age-standardized DALY rate of neglected parasitic diseases in China from 1990 to 2019 and the prediction trend from 2020 to 2030. The prediction results of the model showed that cystic echinococcosis and cysticercosis exhibited an upward trend, while food-borne trematodiases, leishmaniasis, schistosomiasis and soil-derived helminthiasis showed a downward trend.

## 4. Discussion

To the best of our knowledge, this study is one of the most comprehensive reports on the burden of neglected parasitic diseases and their trends in China between 1990 and 2019. Based on the latest and most comprehensive data on neglected parasitic diseases published in GBD 2019, this study analyzed the prevalence, sex and age distribution and disease burden of cystic echinococcosis, cysticercosis, food-borne trematodiases, leishmaniasis, schistosomiasis and soil-derived helminthiasis in China from 1990 to 2019, and used the ARIMA model to predict the trend of each disease’s DALYs from 2020 to 2030, providing essential information for better prevention and treatment of neglected parasitic diseases in China.

Several key observations can be derived from our study. First, Overall, China’s prevalence of neglected parasitic diseases exhibited a downward trend from 1990 to 2019. However, the prevalence of cysticercosis exhibited the most significant increase, and the age-standardized prevalence rate of cystic echinococcosis increased slightly. Second, from the perspective of sex distribution, the total number of cases of neglected parasitic diseases in China was higher in males than in females in 2019. From the perspective of age distribution, the prevalence and disease burden of parasitic diseases in the older adult group was significantly higher. Third, although the age-standardized DALY rate declined from 1990 to 2019, the absolute number of DALYs was still high. The ARIMA model showed that the DALYs of cystic echinococcosis and cysticercosis would increase slightly after 2020 while food-borne trematodiases, leishmaniasis, schistosomiasis and soil-derived helminthiasis would decrease.

In China, the government has made considerable progress in controlling and eliminating neglected parasitic diseases. The Ministry of Health organized and carried out two national surveys of human parasitic diseases in 1988–1992 and 2001–2004, with overall infection rates of 62.63 and 22.53%, respectively. The results of the third national survey on the status of important human parasitic diseases in 2014–2017 showed that compared with the previous two surveys, the infection rate decreased significantly in China below 6%. In 1958, leishmaniasis was nearly eliminated in China, but after entering the 21st century, several outbreaks of leishmaniasis have been observed (14). In 2007, the WHO announced that China was the first country to successfully eliminate lymphatic filariasis (15). In 2008, China achieved the epidemic control goal in endemic schistosomiasis areas (16). In 2020, nearly 97% of schistosomiasis-endemic counties in China had reached the transmission interruption or elimination standard (17). In 2018, 294101 people were screened at the national hydatid disease surveillance points, and 214 cases of Cystic echinococcosis were identified, suggesting an infection rate of 0.07% (18). The third national parasitic disease survey results showed that the overall infection rate of cysticercosis in China was 0.06% with 370000 infected cases, the total infection rate of soil-derived helminthiasis was 3.38%, and the pooled weighted infection rate was 4.49% (19). However, some diseases still can significantly burden our economy, highlighting the need to improve current prevention and control strategies.

From 1990 to 2019, the overall prevalence and disease burden of neglected parasitic diseases in China exhibited a downward trend. Soil-derived helminthiasis accounted for the largest number of cases and the highest DALYs. The prevalence and disease burden in men and older people were higher. Although the age-standardized DALY rate declined from 1990 to 2019, the absolute number of DALYs was still high, substantiating that neglected parasitic diseases remain an important cause of disability and premature death in China.

In terms of sex distribution, the age-standardized prevalence rate and DALY rate of cystic echinococcosis, food-borne trematodiases, schistosomiasis and soil-derived helminthiasis in 2019 in China were higher in men than in women, but the rate of cysticercosis in women was higher than in men. These results suggest a higher risk of disease in men than in women, probably because the relationship between sex and parasitic disease infection risk is often affected by different socio-economic, environmental, occupational and behavioral factors, as well as access to health care services. In this respect, it has been shown that the prevalence of cysticercosis is related to health knowledge and behavior, livestock raising, household toilet structure and fecal disposal (20), and most men go out to work, and more women are engaged in housework such as pig raising at home and have a low awareness of disease prevention (21). In addition, the main patients of schistosomiasis in China are farmers and fishermen, and these jobs are mainly men (22). In some rural areas of China, men have a higher family and social status than women, so men have easier access to health care than women, suggesting that men are more likely to be diagnosed with parasitic diseases and women are unaware of their disease.

From the perspective of age distribution, the prevalence and disease burden of parasitic diseases in the older adult group was significantly higher, which may be caused by the fact that young people go out to work, middle-aged and older people work in household farming, and have increased contact with livestock (23), or because the older adult group often have comorbidities (such as cardiovascular diseases and cancer) and poor immunity, which raise their risk of developing chronic complications from parasitic infections, leading to increased disease severity and mortality. In previous study, the researcher identified aged patients as those exhibiting overall less intense antibody responses, mainly in isotypes/subclasses supposed to exert efficient antiparasitic activities (e.g., IgE and IgG1) (24). Thus, these humoral defects could at least partially explain the reported increase in CE prevalence among older individuals, as a weaker immune response in the older adult might facilitate the establishment and maintenance of the parasite infection. And in another study, the author founded that is probable that helminth infections in the older adult population can intensify the immunosenescence outcomes due to the synergistic immunoregulatory effects of each of them (25). Therefore, more emphasis should be placed on protecting the older adult and increasing the early diagnosis and treatment of such diseases.

Soil-derived helminthiasis is the most serious disease among the neglected parasitic diseases in China. The age-standardized prevalence rate decreased from 241608/100000 in 1990 to 93.702/10000 in 2019, exhibiting the most significant decline. However, many cases of endemic soil-derived helminthiasis are still reported in China, indicating that the prevention and control strategies need further improvement. Food-borne trematodiases remain a public health issue with wide prevalence and difficult diagnosis, and some parasites also yield a carcinogenic effect (26). The prevalence of food-borne trematodiases is closely associated with the consumption of raw and semi-raw food, and the life history of the pathogen is complex, which increases the difficulty of disease prevention and control (27).

Moreover, we found that the number of people suffering from cystic echinococcosis and cysticercosis increased from 1990 to 2019. After age standardization, the prevalence of cysticercosis decreased, while that of cystic echinococcosis increased slightly. This phenomenon may be due to the lack of health resources, systematic and comprehensive prevention, poor treatment of cystic echinococcosis, and the failure to be diagnosed and treated promptly. However, according to the age-standardized DALY rate, the disease burden of cystic echinococcosis exhibited shown a downward trend, which may be attributed to the fact that in 2012, China carried out a survey on cystic echinococcosis, conducted a comprehensive screening of human cystic echinococcosis and promulgated a series of comprehensive preventive and control measures, which exerted a definite effect and reduced the population disability and premature death caused by cystic echinococcosis (28). The age-standardized prevalence rate and DALY rate of schistosomiasis displayed a downward trend from 1990 to 2019, indicating effective control of the epidemic situation of schistosomiasis in China. In 2019, the age-standardized prevalence rate of leishmaniasis was 0, but the age-standardized DALY rate was 19.7, consistent with the literature. However, there are still sporadic reports of leishmaniasis in some regions of China (14).

Disease prediction models, including time series and machine learning, have been developed in recent years. The most commonly used disease prediction model is an autoregressive integrated moving average (ARIMA) (29). In this study, ARIMA was used to predict the disease burden of neglected parasitic diseases in China. The ARIMA model showed that cystic echinococcosis and cysticercosis would increase slightly after 2020 while food-borne trematodiases, leishmaniasis, schistosomiasis and soil-derived helminthiasis would decrease. The model predicted an increase in the burden of cystic echinococcosis and cysticercosis, emphasizing the need for further prevention and control. Since the spread of these two diseases is mainly caused by livestock, the main prevention strategies involve controlling the source of infection, intermediate host prevention and population screening treatment. More emphasis should be placed on health education and encouraging people to have regular physical examinations for disease screening. ARIMA requires time series data to be stable or stable after differential processing. The time series data of most infectious diseases showed non-stationary characteristics, which is a concern of traditional ARIMA models (30). Another limitation of the ARIMA model is the poor accuracy of the results when the observation data sample size is too small. Due to the limited data available for prediction analysis in this study, the accuracy of model prediction needs to be externally verified.

GBD studies provide high-quality estimates of global burden of diseases, yet there exist several limitations. One inevitable limitation is the uncertainty of GBD estimates in cases in which actual data on disease burden are unavailable, and the GBD estimates fill the vacancies in this occasion. Besides, differences in data collecting and coding, as well as quality of data sources, remain inevitable in this analysis pattern. Moreover, the fluctuations in incidence and mortality rates may partly reflect the detection bias related to adjustments in screening protocols instead of real changes in age-specific rates.

## 5. Conclusion

This study harnessed data from the GBD database to analyze the prevalence and burden of neglected parasitic diseases in China from 1990 to 2019 and established a mathematical prediction model. The results showed that the prevalence and disease burden of neglected parasitic diseases in China decreased from 1990 to 2019 but remains relatively high. Our research results provide a scientific basis for further prevention and control measures against parasitic diseases in China. Actively exploring the epidemiological patterns and trends of neglected parasitic diseases is of great significance for formulating targeted disease prevention measures, improving the health status of the general population and preventing disease propagation.

## Data availability statement

The original contributions presented in the study are included in the article/Supplementary material, further inquiries can be directed to the corresponding author.

## Author contributions

YX and DS wrote the manuscript, with contributions from YW and XW. YX and DS conducted the data collection. DS did the analysis. YG critically revised the manuscript. All authors contributed to data interpretation, wrote and revised various parts of the article, and approved the submitted version.

## Conflict of interest

The authors declare that the research was conducted in the absence of any commercial or financial relationships that could be construed as a potential conflict of interest.

## Publisher’s note

All claims expressed in this article are solely those of the authors and do not necessarily represent those of their affiliated organizations, or those of the publisher, the editors and the reviewers. Any product that may be evaluated in this article, or claim that may be made by its manufacturer, is not guaranteed or endorsed by the publisher.

## Footnotes

^1^ https://vizhub.healthdata.org/gbd-results/
