# Supplementary material for: Prevalence trend and burden of neglected parasitic diseases in China from 1990 to 2019: findings from global burden of disease study
Source: Front Public Health. 2023 May 24;11:1077723. doi: 10.3389/fpubh.2023.1077723 (PMC10244527; doi:10.3389/fpubh.2023.1077723)
Supplement: Supplementary file 1 [file Data_Sheet_1.docx]

Supplementary Material

Prevalence Trend and Burden of Neglected Parasitic Diseases in China from 1990-2019: Findings from Global Burden of Disease Study

Yi Xie, Dandan Shi, Xu Wang, Yayi Guan,Weiping Wu, Ying Wang*

*** Correspondence:** Ying Wang: wangying@nipd.chinacdc.cn

# Supplementary Tables

**Table S1 Prevalence and DALYs of 6 neglected parasitic diseases in China in 1990 and 2019 by sex**

| Sex | Items | Prevalence | | | | DALYs | | | |
| --- | --- | --- | --- | --- | --- | --- | --- | --- | --- |
|  |  | No | | ASR | | No | | ASR | |
|  |  | 1990 | 2019 | 1990 | 2019 | 1990 | 2019 | 1990 | 2019 |
| Male | Cystic echinococcosis | 12108.4(3445.7-30551.7) | 17015.4(6772-35642.9) | 1.9(0.6-4.4) | 2.2(0.8-5) | 2845.6(2005.1-4441.1) | 2481.6(1612.1-4077.8) | 0.5(0.4-0.7) | 0.3(0.2-0.5) |
|  | Cysticercosis | 172308.8(115520.1-248968.1) | 294871.9(186804.5-447957.7) | 38.6(26.8-55) | 30.6(20.3-45.6) | 58289.3(34380.7-89309.9) | 73228.1(40352.9-121093.3) | 12.7(7.6-19.1) | 7.6(4.2-12.1) |
|  | Food-borne trematodiases | 20226268(17038775.2-24256031.5) | 16255714.9(13904776.7-19042789.3) | 3597.9(3055.5-4275.3) | 1815.8(1549.4-2116.6) | 549855.5(192340-1167384.4) | 393576(170129.7-770622.8) | 96.4(34-201.7) | 43.8(18.8-86.4) |
|  | Leishmaniasis | 173.4(34-368.8) | 0(0-0) | 0(0-0.1) | 0(0-0) | 58.1(33.7-98.1) | 13.2(7.7-21.3) | 0(0-0) | 0(0-0) |
|  | Schistosomiasis | 8479655.1(7155089.1-11946753.4) | 6154426.5(5395593.7-8113609.7) | 1358.7(1150.6-1919.8) | 783(685.7-1031.5) | 91076.4(59553.7-147069.7) | 44564.7(22447.4-82374.6) | 16.2(11-25) | 5.5(2.7-10.3) |
|  | intestinal nematode infections / Soil-derived helminthiasis | 153369338.6(120424946.1-189563678.3) | 59313220.4(43293787-79195485.1) | 24154.9(18952.8-29724.6) | 9410.1(6845.2-12589.4) | 397007.8(212965.3-664769.2) | 32463.3(17375-56341.8) | 63.1(33.8-105.2) | 5(2.7-8.7) |
| Female | Cystic echinococcosis | 10602.6(3200.9-26066.5) | 14857.9(6571.3-29306.8) | 1.8(0.6-4.1) | 1.9(0.8-4.1) | 2390.2(1610.7-3665.6) | 1777(1086.6-3063.3) | 0.4(0.3-0.6) | 0.2(0.1-0.4) |
|  | Cysticercosis | 213021.8(151547.9-289158.5) | 347269.4(227391.7-498755.6) | 46.5(34-62.7) | 34(22.7-48.7) | 69586.2(42448.8-102035.8) | 83625(47162.4-132581.8) | 14.9(9.2-21.7) | 8.2(4.6-12.8) |
|  | Food-borne trematodiases | 12683084.2(10710835.2-15105083.1) | 10509146.1(9027503.1-12092859.8) | 2374.8(2005.5-2840.2) | 1182.9(1024.1-1362.7) | 334875.1(118080.3-697784.2) | 250260.4(113241.9-482322.6) | 62(22-129.1) | 28(12.6-54.2) |
|  | Leishmaniasis | 0(0-0) | 0(0-0) | 0(0-0) | 0(0-0) | 25.1(14.4-42.6) | 6.5(3.6-11.9) | 0(0-0) | 0(0-0) |
|  | Schistosomiasis | 8088615.7(6856491.8-11337599.2) | 4652976.3(4103481-5997616.3) | 1338.4(1142.1-1893.1) | 628.4(555.4-808.4) | 108541.5(72066.2-166806.5) | 35199.9(18007.8-64954.5) | 18.8(12.7-28) | 4.6(2.3-8.6) |
|  | intestinal nematode infections / Soil-derived helminthiasis | 144395401(113360692.3-178052448.7) | 54958563.6(40183451.4-73026940) | 24187.9(19013.8-29730) | 9328.8(6800.2-12452.5) | 466169.2(257581.8-765358.2) | 38526.4(21296.2-64929.1) | 75.9(42.1-123.9) | 6.1(3.4-10.3) |

# Supplementary Figures


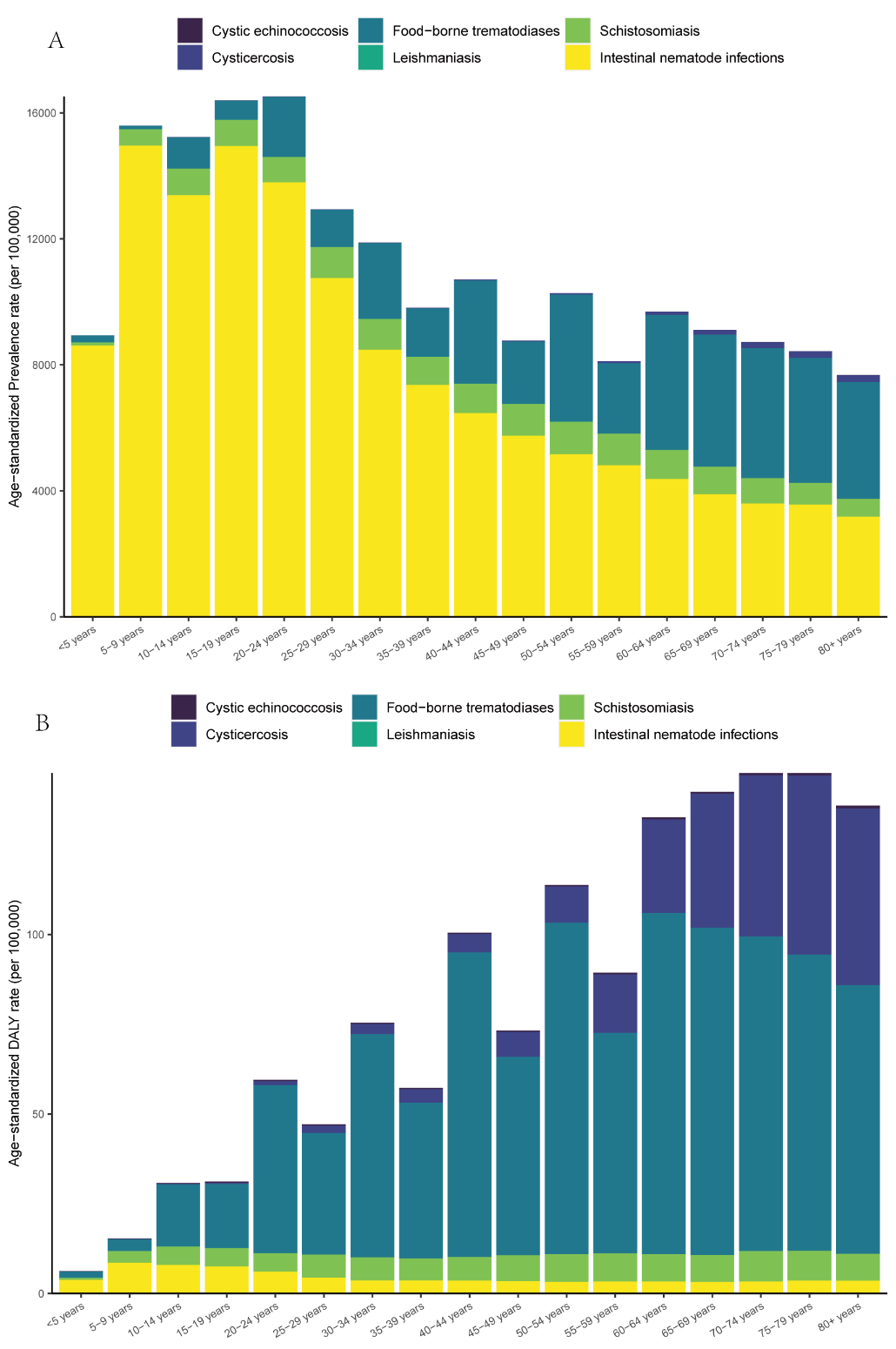


Figure S1 Age-standardized prevalence and DALY rate of neglected parasitic diseases in male in China by age in 2019


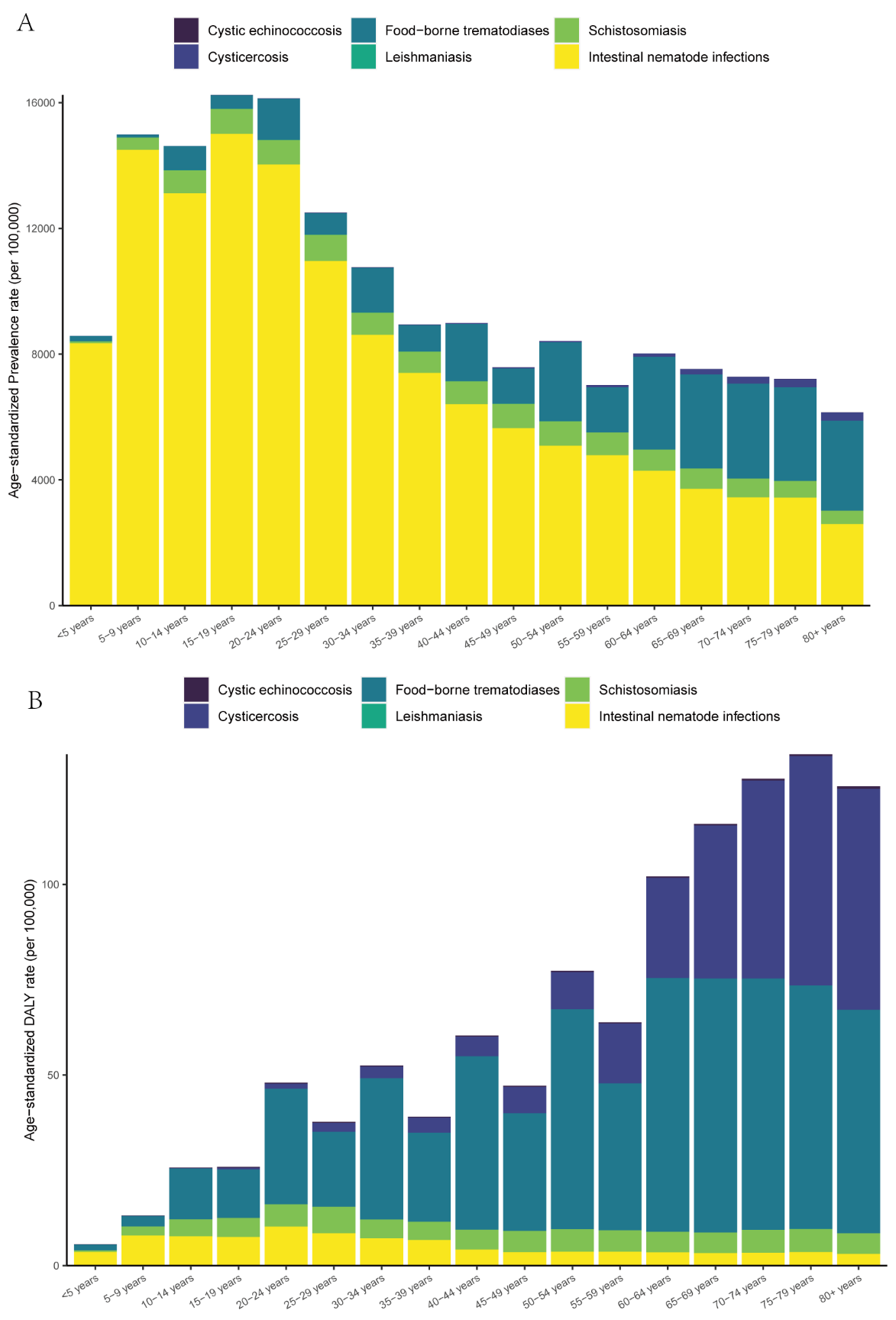


Figure S2 Age-standardized prevalence and DALY rate of neglected parasitic diseases in female in China by age in 2019
